# The Value of Disinfectants

**Published:** 1921-10

**Authors:** 


### The Value of Disinfectants.

*To the Editor of* THE HOSPITAL AND HEALTH REVIEW.

SIR,—I should like to hear the opinion of some of your readers about the value of disinfectants. Are they any good at all, or might we not just as well pour our money down the drain? Of course, I think that steam disinfection is very useful, but are any of the other ways of disinfecting any good to prevent the spread of disease, or are they only "eye-wash"? I enclose a stamped addressed envelope so that you may send me any replies that you do not want to publish.—I am, yours, &c.,

A RURAL MEDICAL OFFICER.

P.S.—I am not asking about disinfectants and their use in test-tubes in laboratories. What I want to know is: Are they any good in a country cottage where there has been, let us say, a case of small-pox?

